# Systems Pharmacological Approach to Investigate the Mechanism of *Ohwia caudata* for Application to Alzheimer’s Disease

**DOI:** 10.3390/molecules24081499

**Published:** 2019-04-17

**Authors:** Yi-wei Sun, Yue Wang, Zi-feng Guo, Kai-cheng Du, Da-li Meng

**Affiliations:** School of Traditional Chinese Materia Medica, Shenyang Pharmaceutical University, Shenyang 110016, China; 18609881535@163.com (Y.-w.S.); 13324242817@163.com (Y.W.); 18179788424@163.com (Z.-f.G.); kaichengdu@163.com (K.-c.D.)

**Keywords:** *Ohwia caudata*, network pharmacology, Alzheimer’s disease

## Abstract

*Ohwia caudata* (OC)—a traditional Chinese medicine (TCM)—has been reported to have large numbers of flavonoids, alkaloids, and triterpenoids. The previous studies on OC for treating Alzheimer’s disease (AD) only focused on single targets and its mechanisms, while no report had shown about the synergistic mechanism of the constituents from OC related to their potential treatment on dementia in any database. This study aimed to predict the bioactive targets constituents and find potential compounds from OC with better oral bioavailability and blood–brain barrier permeability against AD, by using a system network level-based in silico approach. The results revealed that two new flavonoids, and another 26 compounds isolated from OC in our lab, were highly connected to AD-related signaling pathways and biological processes, which were confirmed by compound–target network, Gene Ontology (GO) analysis, and Kyoto Encyclopedia of Genes and Genomes (KEGG) pathway enrichment analysis, respectively. Predicted by the virtual screening and various network pharmacology methods, we found the multiple mechanisms of OC, which are effective for alleviating AD symptoms through multiple targets in a synergetic way.

## 1. Introduction

Alzheimer’s disease (AD) is a progressive neurodegenerative disorder with age-dependent memory dysfunction, affecting mainly the elderly, and its prominent feature is progressive memory dysfunction together with a number of cognitive impairments and bodily functions deteriorations [1,2,3]. According to a statistical report, the number of patients was predicted to be over 100 million by 2050 [4,5]. Preventing and treating AD become imperative in contemporary clinical therapy around the world, but the key pathogenesis can hardly be understood due to the multiple mechanisms, such as accumulation of amyloid beta (Aβ), neurofibrillary tangles (NFTs), metabolic disturbances, as well as oxidative stress [6,7,8].

The multiple mechanisms involved in the pathogenesis of AD create considerable difficulties in developing an effective treatment. Currently, the FDA-approved anti-AD drugs (donepezil, galantamine, rivastigmine, and memantine) are of mainly two types: acetyl cholinesterase inhibitors and *N*-methyl-d-aspartate receptor antagonist [9,10]. However, these drugs only provide a symptomatic, palliative pharmacological effect which will wears off after a certain treatment time. The efficacy of single targeting therapies only helps to produce symptomatic pharmacological effect rather than efficient disease-modifying effects [11]. In this situation, new anti-AD drugs with multitarget-directed properties are in urgent need.

Considering the complexity of the mechanisms involved in AD, traditional Chinese medicines (TCMs) provide potential for promoting development of AD therapy with multicomponent and multitarget synergistic therapeutics. Besides, botanicals are important sources of natural biological compounds and potential leading drugs, especially for complicated diseases [12,13]. *Ohwia caudata* is an evergreen plant belonging to the family of Fabaceae, distributed in south of China, India, and Japan [14]. Its stems and roots have been used medicinally to treat various diseases like fever, dysentery, icterohepatitis, and abscess [15]. In a previous report, the flavonoids in *O. caudatum* exhibited free radical scavenging abilities and anti-Aβ aggregation effects, which revealed the close associations between *O. caudatum* and anti-AD biological activities [16]. However, the biological activities of alkaloids, triterpenoids, and other phenolics from this plant have not been studied in depth. On the other hand, the blood–brain barrier is anatomically characterized by the presence of intercellular tight junctions between continuous nonfenestrated endothelial cells, which normally functions to limit the passage of macromolecular compound into the brain parenchyma [17]. Although the drugs have strong anti-AD activity, most of leading drugs fail in clinical trials due to poor absorption, distribution, metabolism, and excretion (ADME) properties and blood–brain barrier (BBB) penetration [18]. Herein, further computational prediction of pharmacokinetic parameters and ADME and BBB analyses have filtered active compounds in OC for the treatments of central nervous system diseases. Through the network pharmacology approach, we explored the potential targets for treating AD and established the compounds–targets–AD network, which provides valuable insight into the efficiency of OC for the prevention of AD.

## 2. Results

**Figure 1:**
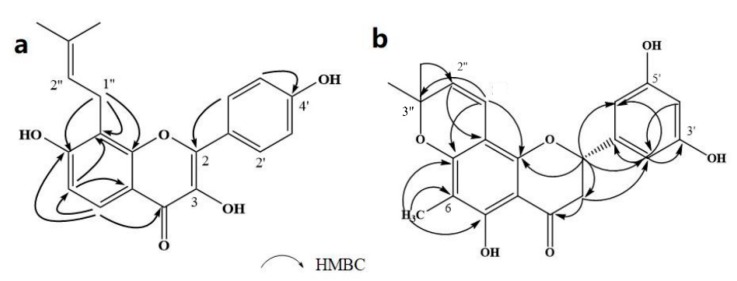
Structures of compounds: (**a**) Compound **1** and (**b**) Compound **2**.

Compound **1** was obtained as a yellow amorphous powder with a molecular formula of C_20_H_18_O_5_ as determined by the high-resolution electrospray ionization mass spectrometry (HRESIMS) at *m*/*z* 337.1076 [M − H]^−^ (calcd. for 337.1098). The ^1^H- and ^13^C-NMR spectra were correlated to those of noranhydroicaritin (C17) except for the absence of a hydroxyl hydrogen signal at H-5, but instead two ortho-hydrogen proton signals (δ_H_ 7.80 ((1H, d, *J* = 8.8 H), δ_H_ 6.97 (1H, d, *J* = 8.8 Hz)) were observed. The ^13^C-NMR spectra of compound **1** showed 20 signals due to two benzene rings, a prenyl group, two oxygen-bearing sp^2^ carbons, and a carbonyl group, in each case. These spectroscopic data indicated that compounds **1** was a flavonol derivatives with a prenyl group either at C-6 or C-8. The location of the prenyl group of **1** was assigned to be at C-8 from the HMBC correlations of the methylene proton (δ_H_ 3.56 (2H, d, *J* = 6.4 Hz)) of the prenyl group with two oxygen-bearing sp^2^ carbons δ159.3 (C-7) and 154.1 (C-9), one of which also had an HMBC correlation with the chelated hydroxyl proton. On the basis of this observation, the data and the structure of **1** were established as shown in Table 1 and Figure 1a.

Compound **2** was obtained as a yellow amorphous powder with a molecular formula of C_21_H_20_O_6_ as determined by the HRESIMS at *m*/*z* 369.1358 [M + H]^+^ (calcd. for 369.1338). The ^1^H- and ^13^C-NMR spectra were well correlated with those of desmodin B (C18). However, the signals were single peak and no small coupling constant values (δ_H_ 6.90 (1H, s), δ_H_ 6.75 (2H, s)) were observed in the aromatic signals in B-ring of compound **2**, which means the presence of a 2,4,6-*tri*-substituted benzene ring. The HMBC correlations of the H-1″ with C-7, C-8, and C-9 indicated that the 2,2-dimethyl-2*H*-pyran ring was attached to C-7 and C-8, while the location of the methyl group was concluded to be at C-6 from the HMBC correlations of the methyl signal with C-5, C-6, and C-7. The absolute configuration at the C-2 was assigned as 2*R* by the CD spectral analysis, in which the positive cotton effect at 325 nm and the negative one at 286 nm were similar to those of the related compounds. On the basis of this observation, the data and the structure of **2** were characterized as shown in Table 1 and Figure 1b.

### 2.1. Identification of Active Compounds

Among 63 compounds isolated from OC, 28 compounds were selected for their pharmaceutically significant ADME properties by using QikProp v3.0 tool of Schrodinger software (Schrödinger Inc., New York, NY, USA). These properties are

1. Aqueous solubility (QPlogS) (−6.5–0.5)

2. Caco-2 cell permeability in nm/sec (<25 poor, >500 great)

3. Brain/blood partition coefficient (QPlogBB) (−3.0–1.2)

4. Apparent Madin–Darby canine kidney (MDCK) cell permeability (QPPMDCK) (<25 poor, >500 great)

5. Percent human oral absorption (≥80% is high, ≤25% is poor)

6. Rule of five (maximum is 4)

7. Rule of three (maximum is 3)

The ADME values of selected compounds were given in Table 2 and their names and structures were shown in Table 3. The five rules are molecular weight < 500, octanol/water partition coefficient < 5, estimated number of hydrogen bonds that would be donated by the solute to water molecules in an aqueous solution ≤ 5, and estimated number of hydrogen bonds that would be accepted by the solute from water molecules in an aqueous solution ≤ 10. Compounds that meet all of the requirements above are considered as drug-like. The three rules are: solubility QPlogS > −5.7, QPPCaco (Caco-2 cells are a model for the gut–blood barrier) > 22 nm/s, primary metabolites < 7. Compounds that satisfy these rules are more likely to be orally available. Brain/blood partition coefficient (QPlogBB) parameter indicated about the ability of the drug to pass through the blood–brain barrier which is mandatory for inhibition. The QPPMDCK predicted apparent MDCK cell permeability in nm/s. MDCK cells are considered to be a good mimic for the blood–brain barrier. All ADME properties showed by selected compounds are in acceptable range.

### 2.2. Compound–Target Network

**Figure 2:**
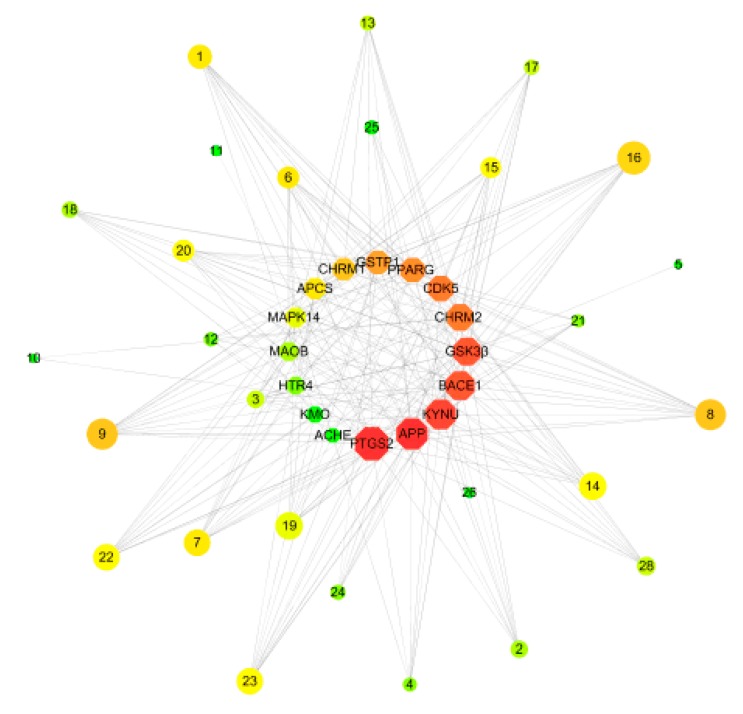
Compound–target network for potential inhibitors in OC. There is a positive correlation between the area of compounds and targets.

Multifactorial mechanisms of AD have been proposed previously, which indicated that more than one hypothesis is involved in the pathogenesis of AD, such as amyloid cascade, tau, neuroinflammation, oxidative stress, and glutamate system dysfunction [19,20]. Thus, 16 targets related to different AD pathogenesis were selected to determine the main pathway of anti-AD effect of OC. The drug–target network was built as shown in Figure 2. *N*,*N*-dimethyltryptamine *N*12-oxide (**C8**, degree = 15), C4-hydroxy-3-methoxyphenyl-β-d-glucopyranoside (**C15**, degree = 15), ferulic acid (**C14**, degree = 13), and *N*-chloromethyl-*N*,*N*-dimethyltryptamine (**C9**, degree = 12) might play an important role in the treatment of AD. PTGS2 (degree = 19), Kynureninase (degree = 19), CHRM2 (degree = 18), and BACE1 (degree = 18) and CdK5 (degree = 18) might be the hub target of this network. Twenty-eight active compounds connected with more than two targets and all targets interact with more than one compound, indicating that most of compounds displayed multitarget-directed properties in treating AD.

### 2.3. Compounds–Target–Target Network

**Figure 3:**
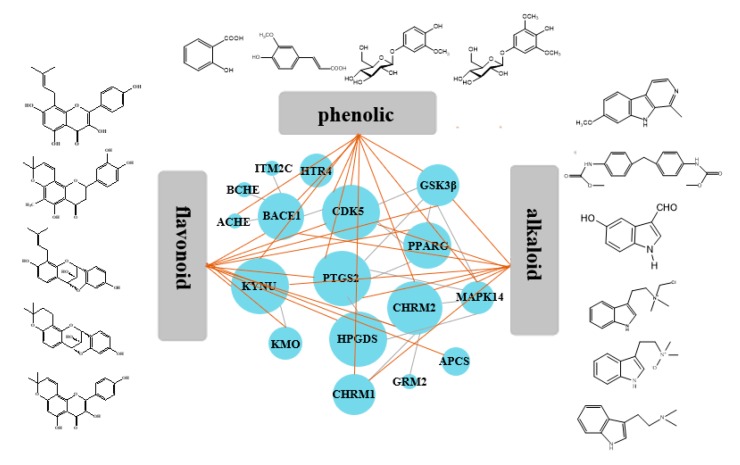
Analyses of compound–target–target (C–T–T) interaction. The 3 gray areas represent compound types and 16 blue circles represent the targets of OC.

Then, based on the results of STRING 10.5 about the interaction of targets, we built a “Target–Target Network” as descripted in Figure 3. Compounds with eight or greater targets were screened out for structural analysis. PTGS2 and KYNU were the center targets in this network and possessed the most edges. In addition, CdK5, BACE1, CHRM2, and GSTP1 were also important nodes. With respect to structures, the alkaloid compounds and phenolic compounds with benzene rings have more targets than heterocyclic rings compounds. Isopentenyl flavonoids have more AD-related targets than triterpenoids and lignans.

### 2.4. GO Analysis

**Figure 4:**
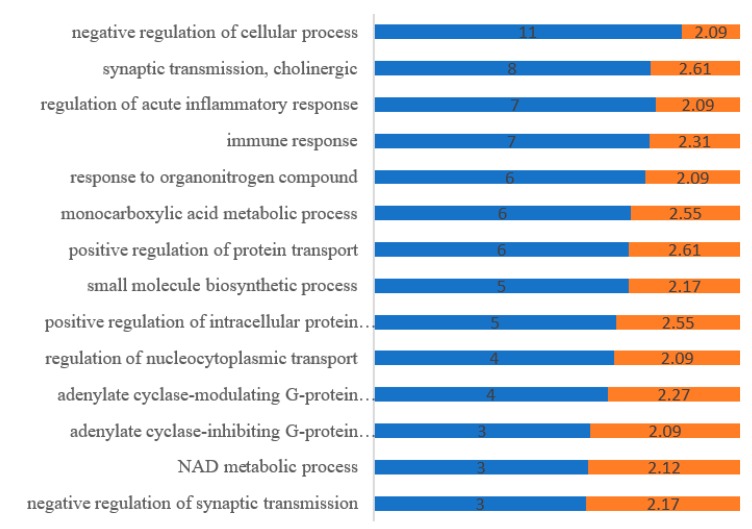
Gene Ontology (GO) biological process (GOBP) analysis. Counts of genes (blue) and −log10 *p*-value (orange) related to each biological process from DAVID 6.8 database.

GO biological process (GOBP) describes a series of events accomplished by one or more organized assemblies of molecular function. GOBP showed that these targets were enriched to 20 biological process terms and all them are highly related to negative regulation of cellular process, synaptic transmission, immune response, immune response positive regulation of protein transport, and so on (as shown in Figure 4).

### 2.5. Compound–Target–Pathway Network

**Figure 5:**
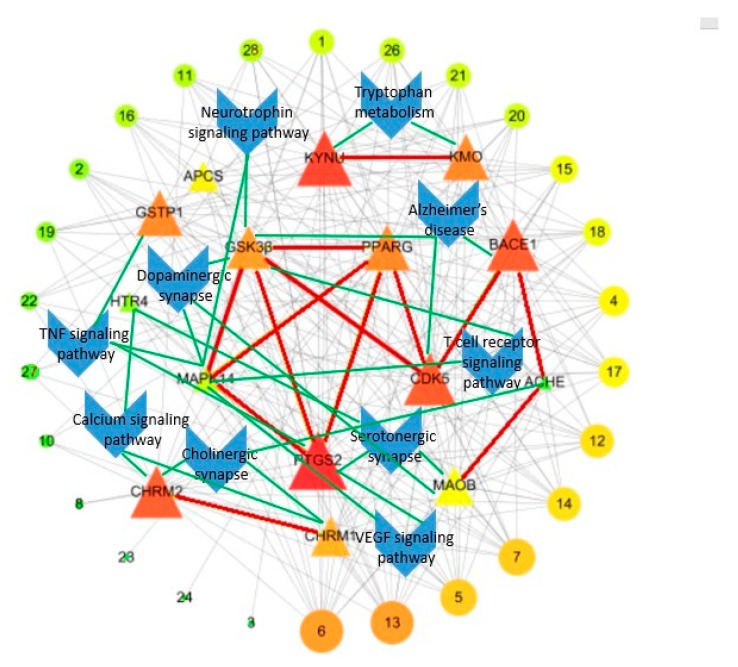
Compound–target–pathway network (C–T–P); where triangle nodes represent the targets, circular nodes represent the compound, and blue nodes represent the pathways.

Based on the prediction of KEGG by DAVID 6.8, the compound–target–pathway network was generated by connecting potential pathways and corresponding targets. Multiple AD related pathways depicted in Figure 5 revealed possible mechanism involved in OC for AD treatment. The key features of this network were tryptophan metabolism, cholinergic synapse, dopaminergic synapse and serotonergic synapse. Some other signal pathways, such as calcium signaling pathway, TNF signaling pathway, T cell receptor signaling pathway, VEGF signaling pathway, and neurotrophin signaling pathway had been known to be associated with AD treatment.

All the targets mentioned above were mapped to KEGG database, and the results were analyzed and sorted. From Figure 5, it is preliminarily speculated that the above compounds could be used for the treatment of AD through two pathways due to the highly correlation with KYNU and PTGS2 targets.

### 2.6. Kynurenine Pathway

Kynureninase, an enzyme exists in the kynurenine pathway, is essential for tryptophan metabolism to yield 3-hydroxyanthranilate leading to the de novo biosynthesis of NAD^+^. This pathway results in 3-hydroxyanthranilic and leading to the formation of quinolinate. Quinolinate is a neurotoxic NMDA receptor antagonist and potential endogenous inhibitor of NMDA receptor signaling in axonal targeting, synaptogenesis and apoptosis, which is highly correlated with the Alzheimer’s disease [21,22]. According to Figure 3, alkaloid compounds such as **C3**, **C8**, and **C9** had the strongest interaction with KYNU indicated that those alkaloids in OC should be considered for the designing and screening novel kynureninase inhibitors.

### 2.7. Inflammation-Related Pathways

PTGS2 is responsible for production of inflammatory prostaglandin from arachidonic acid and plays important roles in neuroinflammation. The presence of inflammation has been identified in the hippocampus of the brains of patients afflicted with AD, performance for neurofibrillary tangles, and neuritic plaques [23]. Flavonoids with isopentyl groups were highly correlated with PTGS2 instead of MAPK 14, which indicated that they could reduce inflammation in neuronal by suppressing the production of inflammatory prostaglandins.

The other pathways, such as calcium signaling pathway and neurotrophin signaling pathway, were also closely related to AD. We found that phenolic glycosides, 4-hydroxy-3-methoxyphenyl-β-d-glucopyranoside (**C15**), and koaburaside (**C16**) were highly targeted to CHRM1 and CHRM2. Two tetracyclic flavans—caudatan C (**C22**) and caudatan A (**C25**)—were targeted to BACE1. Most flavonoids were targeted with CdK5.

### 2.8. Molecular Docking

Docking studies were performed using Molegro Virtual Docker in the active sites of five hub targets in order to investigate the possible interactions between the compounds and the active site of the targets, namely, PTGS2 (PDB code 5F19), KYNU (PDB code 2HZP), CHRM2 (PDB code 3UON) BACE1 (PDB code 3UQU) and CdK5 (PDB code 3O0G). The docking scores were depicted in Table 4.

Before docking, the X-ray structure of localization inhibitors in PTGS2 (Protoporphyrin IX containing CO), KYNU (Pyridoxal-5′-phosphate), CHRM2 ((3*R*)-1-azabicyclo[2.2.2]oct-3-yl hydroxy(diphenyl)acetate), BACE1 (*N*-[(1*R*)-1-(4-fluorophenyl)ethyl]-*N*′-[(2*S*,3*S*)-3-hydroxy-1-phenyl-4-(1*H*-pyrazol-1-yl)butan-2-yl]-5-[methyl(methylsulfonyl)amino]benzene-1,3-dicarboxamide), and CdK5 ({4-amino-2-[(4-chlorophenyl)amino]-1,3-thiazol-5-yl}(3-nitrophenyl)methanone) were taken from the PDB. Thus, root mean square deviations (RMSDs) of proteins cocrystalized with localization inhibitors were 1.143 Å, 0.789 Å, 0.478 Å, 1.150 Å, and 0.993 Å, respectively, which demonstrated that the docking procedure could be relied to predict the binding mode of our compounds.

**Figure 6:**
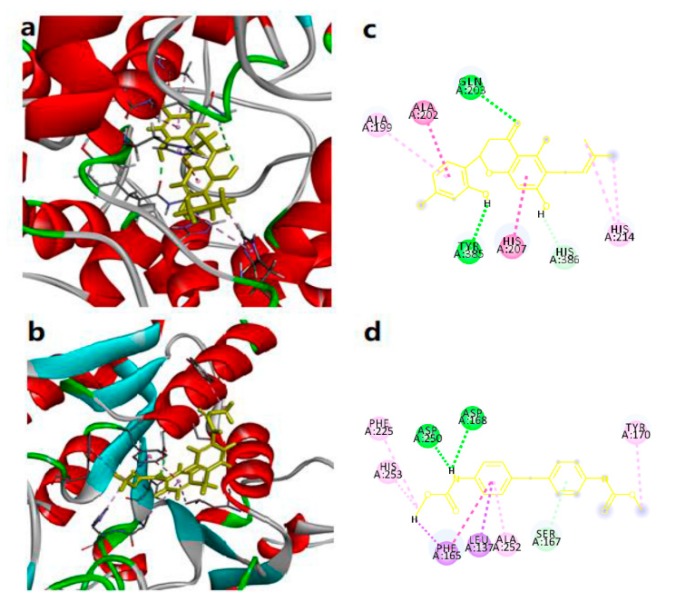
(**a**) Molecular model of most active molecule in the compound 19 in the protein PTGS2 (protein data bank ID chimeric 5F19). Active site amino acid residues are represented as tubes, while the inhibitor is shown as stick model with yellow colored. (**b**) Molecular model of most active molecule in the compound 4 in the protein KYNU (protein data bank ID chimeric 2HZP). (**c**) Schematic (2D) representation of interactions of compound 19 in the binding pocket of the protein. (**d**) Schematic (2D) representation of interactions of compound 4 in the binding pocket of the protein.

The binding mode of compound **19** in the active site of PTGS2 had been represented in its three-dimensional mode in Figure 6a, while the schematic 2D dimensional representation had been shown in Figure 6c. **C17** showed two H-bond interactions—one was a carbonyl group from the flavonoid skeleton with GLN 023 residue. The second one was the C-OH group presented in the phenolic hydroxyl group on the B ring of flavonoid linked with TYR 385 residue. The isopentenyl side chain linked to HIS 214 residue and the phenyl rings interacted with the side chain of ALA 202, HIS 207 and HIS 214. The other key residues which involved in interaction were PHE 225, HIS 253, PHE 165, TYR 170, ALA 252, and LEU 137. However, this score was found to be lesser than the docking score of the reference ligand.

The binding mode of compound **4** in the active site of KYNU is represented in its three-dimensional mode in Figure 6b, while the schematic 2D dimensional representation is shown in Figure 6d. **C2** showed two H-bond interactions between the N-H group with ASP 168 and ASP 250 residue. The other key residues which involved in interaction were PHE 225, HIS 253, PHE 165, TYR 170, ALA 252, and LEU 137. These interactions increased the binding affinity of the molecule as indicated by the docking score of the compound **4** as −112.357, which is comparable and far more than the dock score −71.135 of the reference ligand.

## 3. Discussion

The network pharmacology approach is developed to discover new therapeutic directions for drugs in natural products from the perspective of molecular biological network. Hence, it provides systematic means to extend the druggable compounds in TCM applied in various unexplored complex diseases [24,25].

In the study, we have evaluated the active compounds and potential targets from OC against AD based on a systematic pharmacological method, including ADME system assessment; drug targeting, mechanism, and pathway research; and molecular docking. Twenty-eight active compounds were detected and interacted with 16 different targets associated with AD. According to the analysis of the C–T network model, **C8** and **C15** exhibited the largest number of targets connections (**15**), followed by **C9** and **C14** (**12**). These high-degree nods play a dominant role in the anti-AD system.

PTGS2 and KYNU targets are the key factors in the drug–target interaction network. There were also plenty of compounds from OC, which were potential inhibitors for CHRM2, CdK5, and BACE1. Although there were several active flavones, alkaloids and phenols could interact with multiple targets, and the binding ability with different skeletons were not necessarily same. For example, alkaloid compounds such as **C3**, **C8**, and **C9** had the strongest interaction than flavonoids with KYNU while the opposite is true in PTGS2. We suggest that compounds and targets with high degree and betweenness values, are the key point in treating AD.

Combined with molecular docking results, the alkaloids with aryl rings may serve as a prominent scaffold for exploring latent KYNU inhibitors. Meanwhile, these flavonoids substituted particularly with isopentyl group may have greet effect on AD from the perspective of anti-inflammatory.

These studies indicate that OC is characterized as a multicompound content, multiobjective regulation, and multipathway cooperation to treat AD. The response to inflammatory, immune, memory, and neuroactive interaction mechanisms of OC is illustrated by analyzing compound–target–pathway network. KYNU and PTGS2 had the highest degree of compound–target, which indicated that kynurenine pathway and inflammation-related pathways possessed synergistic or additive anti-AD effect. Indeed, CdK5, BACE1, and CHRM2 are somewhat connected to the neuron protecting, synapse part, and may inhibit Aβ aggregation and calcium signaling dysfunction through calcium signaling pathway and G-protein coupled receptor signaling pathway.

In conclusion, to the best of our knowledge, we were the first to elucidate the mechanisms of action for OC on AD treatment, through the virtual screening with systems pharmacological approach.

## 4. Materials and Methods

### 4.1. Plant Material

*Ohwia caudata* (Thunb.) H. Ohashi. was collected from Huai Hua, Hu Nan province, China (lat. 27°31′56″N, long. 110°0′20.64″E; altitude 240 m a.s.l.) at a dry season in June 2013, and was identified by Prof. JinCai Lu at Shenyang Pharmaceutical University.

### 4.2. Establishment of Database

There is no database containing compounds from OC. Therefore, all the compounds of OC were collected from the leaves, stems and roots of *Ohwia caudata* isolated in our lab. Two dimensional (2D) structures of the compounds were sketched using Chembiodraw 2014 (CambridgeSoft, Cambridge, MA, USA).

### 4.3. Extraction and Isolation

The air-dried stems of *O. caudatum* (10.0 kg) were chopped into small pieces and extracted with 70% aqueous EtOH (200 L) under reflux for 4 h. After evaporation of the combined EtOH extracts in vacuo, the resultant residues (1.2 kg) were suspended in water and subjected to macroporous adsorptive resin (HPD 100, Cangzhou Bon adsorber Technology Co., Ltd., Cangzhou, China) column chromatography to elute sequentially with H_2_O, 40%, 60%, and 95% EtOH, respectively. The 95% eluates (65 g) were chromatographed on silica gel column (500 mm × 100 mm i.d.) using a gradient CH_2_Cl_2_-MeOH system (100:0–0:100, *v*/*v*) to give seven fractions (1–7). Fr. 3 (2.2 g) was also purified by silica gel column chromatography (220 mm × 60 mm i.d.) eluting with petroleum ether–acetone (50:1–1:1, *v*/*v*) and the Sephadex LH-20 (GE Healthcare, Uppsala, Sweden) column (500 mm × 15 mm i.d.) eluting with methanol successively, and further separated by preparative RP-HPLC using CH_3_CN/H_2_O as elution solvent to give compounds **1** (12.9 mg). Fr. 5 was separated by using silica gel CC eluting with PE/A (50:1–3:1, *v*/*v*), Sephadex LH-20 column eluting with MeOH and preparative (RP-HPLC) using MeOH/H_2_O as eluting solvent to afford compounds **2** (8.3 mg). All solvents used were analytically pure. The NMR spectral data of compounds **1** and **2** are available in Supplementary Materials.

### 4.4. Prediction of Drug-Likeness, Oral Bioavailability, and Blood–Brain Barrier Permeability

There are various kinds of compounds contained in OC, including flavonoids, alkaloids, triterpenoids, and phenolics, but only bioactive compounds can contribute to clinical treatment. Thus, prior to the target prediction, compound which have good ADME and BBB properties is an important aspect of drug discovery. To streamline the virtual screening, ADME properties of all the 65 compounds were predicted to select active compounds using QikProp, version 3.0 of Schrodinger [26]. QikProp provides ranges for comparing properties of a particular molecule with 95% of known drugs. It also flags 30 types of reactive functional groups that may cause false positives in high-throughput screening (HTS) assays [27].

### 4.5. Target Fishing

BATMAN-TCM (http://bionet.ncpsb.org/batman-tcm/), Therapeutic Target Database (TTD) (http://bidd.nus.edu.sg/BIDD-Databases/TTD/TTD.asp) and TCMSP database (http://lsp.nwu.edu.cn/browse.php?qc=herbs) were employed for protein targets prediction. The target names of the focused proteins were uniformly standardized and downloaded from the RCSB Protein Data Bank (RCSB PDB) database (http://www.rcsb.org/pdb/home/home.do). In consequence, 16 target proteins were obtained by searching the PDB database.

### 4.6. Compound–Target Network Construction

The network pharmacology is extensively used to identify the possible targets of natural products. The “compound–target network” is a direct interactive network which is composed of node and edge by linking candidate compounds and targets. The active compounds–targets network is established by CytoScape v3.4.0 (https://cytoscape.org/) [28].

### 4.7. Compounds–Target–Target Network

The active “compounds–target–target network” is established based on STRING (Search Tool for the Retrieval of Interacting Genes/Proteins, http://string-db.org/) analysis [29].

### 4.8. Gene Ontology (GO) Analysis

The Gene Ontology (GO) project is a major bioinformatics initiative to develop a computational representation of our evolving knowledge of how genes encode biological functions at the molecular, cellular, and tissue system levels. It classifies functions into three aspects: molecular function (molecular activities of gene products), cellular component (where gene products are active), and biological process (pathways and larger processes made up of the activities of multiple gene products) [30]. In this study, GO terms with *p*-values < 0.01 and Benjamini < 0.05 were employed and the data were collected by the DAVID 6.8 (Database for Annotation, https://david.ncifcrf.gov/) prediction.

### 4.9. Compound–Target–Pathway Network

KEGG pathway enrichment analysis provides not only pathway functional annotations of given gene set but also pathway enrichment analysis. Based on the results in DAVID database, the Cytoscape 3.4.0 software was used to construct the compound–target-pathway network as shown in Figure 4. The characteristics of multiple components, multiple targets and multiple pathways of OC were revealed through the construction of network.

### 4.10. Molecular Docking

As a kind of in silico target prediction tool, molecular docking has been widely used as ligand-based target prediction and structured-based target prediction. At present, this virtual screening is a promising way to identify putative targets for a specific ligand. To evaluate these targets, the crystal structures of candidate targets were downloaded from RCSB Protein Data Bank (http://www.pdb.org/) [31] and embellished through the Sybyl-X (version 2.0, TRIPOS Inc., St. Louis, USA) software, including removing the ligands, adding hydrogen, removing water, and optimizing and patching amino acids. Before docking, ChemBioDraw 3D was used to make three dimensional chemical structural formulas and energy minimizing for all the compounds, then saved results in MOL.2 format. Moreover, a suitable method used to evaluat the precision of a docking procedure is needed. The accuracy and consistency of the docking results model obtained by Molegro Virtual Docker (MVD). Briefly, the best docking poses between the predicted conformation and the observed X-ray crystallographic conformation were compared and denoted by the root mean square deviations (RMSDs). A model can be considered as reliable or accurate model when its RMSD is less than 3 Å (accurate ≤ 2 Å, reliable ≤ 4 Å) [32].

## Figures and Tables

**Table 1:** Data for Compounds **1** and **2** in DMSO-*d*_6_ (600 MHz for ^1^H-NMR, 150 MHz for ^13^C-NMR).

No.	Comp. 1 δ*_C_*	Comp. 1 δ_H_	Comp. 2 δ_C_	Comp. 2 δ_H_
2	145.2		78.4	5.43 (1H, dd, *J* = 12.2, 2.8 Hz)
3	137.0		42.0	3.21 (1H, dd, *J* = 12.2, 17.0 Hz) 2.76 (1H, dd, *J* = 2.8, 17.0 Hz)
4	172.3		197.2	
5	123.4	7.80 (1H, d, *J* = 8.8 Hz)	159.5	
6	113.9	6.97 (1H, d, *J* = 8.8Hz)	104.0	
7	159.4		158.9	
8	114.4		102.0	
9	154.1		154.7	
10	114.5		101.0	
1′	122.6		129.5	
2′	129.3	8.03 (1H, d, *J* = 8.9 Hz)	115.4	6.75 (1H, s)
3′	115.5	6.93 (1H, d, *J* = 8.9Hz)	145.3	
4′	158.8		117.7	6.90 (1H, s)
5′	115.5	6.93 (1H, d, *J* = 8.9Hz)	145.7	
6′	129.3	8.03 (1H, d, *J* = 8.9 Hz)	114.2	6.75 (1H, s)
1″	21.9	3.56 (2H, d, *J* = 6.6 Hz)	115.3	6.44 (1H, d, *J* = 10.0 Hz)
2″	122.1	5.22 (1H, t, *J* = 6.6 Hz)	126.7	5.63 (1H, d, *J* = 10.0 Hz)
3″	131.5		77.9	
4″	25.9	1.77 (s, 3H)	28.0	1.38 (s, 3H)
5″	17.9	1.63(s, 3H)	27.9	1.41((s, 3H)
6-Me			7.4	1.89 (s, 3H)

**Table 2:** Prediction of poor absorption, distribution, metabolism, and excretion (ADME) and blood–brain barrier (BBB) properties using QikProp.

Comp.	QPlogS	QPPCaco	QPlogBB	QPP MDCK	Percent Human Oral Absorption	Rule of Five	Rule of Three
**C1**	−4.993	287.747	−1.28	128.708	86.403	0	0
**C2**	−5.733	178.660	−1.35	76.892	84.863	0	1
**C3**	−3.294	4887.536	0.23	2749	100	0	0
**C4**	−5.055	337.098	−1.34	152.725	90.744	0	0
**C5**	−0.393	514.138	−0.52	241.025	73.539	0	0
**C6**	−1.542	328.798	−0.76	148.665	78.23	0	0
**C7**	−1.08	2646.027	−0.133	1416.199	100	0	0
**C8**	−1.08	2646.103	−0.13	1416.243	100	0	0
**C9**	−1.881	1220.263	0.50	678.697	96.387	0	0
**C10**	−0.22	497.093	0.61	516.978	80.741	0	0
**C11**	1.168	1710.644	0.17	1785.53	90.664	0	0
**C12**	−1.756	1202	−0.22	603.556	87.474	0	0
**C13**	−1.554	116.972	−0.62	61.872	77.047	0	0
**C14**	−1.694	97.467	−0.99	50.8	70.752	0	0
**C15**	−1.171	192.969	−1.44	83.57	62.799	0	0
**C16**	−1.228	223.474	−1.47	97.936	64.567	0	1
**C17**	−4.368	125.867	−1.72	52.658	79.335	0	1
**C18**	−5.121	216.221	−1.26	94.505	86.586	0	0
**C19**	−4.656	159.964	−1.65	68.233	82.202	0	1
**C20**	−3.685	127.628	−1.52	53.454	78.142	0	1
**C21**	−4.863	196.428	−1.26	85.19	85.179	0	0
**C22**	−3.737	457.384	−0.95	212.4	90.08	0	1
**C23**	−4.444	220.66	−1.23	96.604	84.383	0	0
**C24**	−4.489	232.352	−1.23	102.148	83.481	0	0
**C25**	−4.637	1114.163	−0.481	556.027	100	0	0
**C26**	−5.976	1562.723	−0.49	801.518	100	1	1
**C27**	−6.127	1015.613	−0.60	503.063	100	0	1
**C28**	−3.871	640.678	−1.09	305.739	93.583	0	1

**Table 3:** 28 potential active compounds of *Ohwia caudata* (OC).

No.	Name	Structure	No.	Name	Structure
**C1**	compound **1**	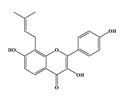	**C15**	4-hydroxy-3-methoxyphenyl-β-d-glucopyranoside	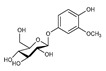
**C2**	compound **2**	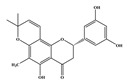	**C16**	koaburaside	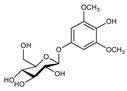
**C3**	harmine	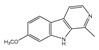	**C17**	noranhydroicaritin	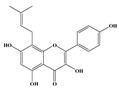
**C4**	4,4′-diphenylmethane-bislmethy carbamate	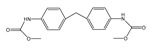	**C18**	desmodin B	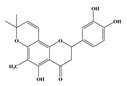
**C5**	nicotinamide	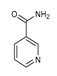	**C19**	cudraflavanone B	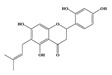
**C6**	5-hydroxy-indole-3-aldehyde	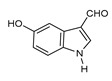	**C20**	leachianone G	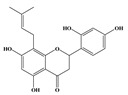
**C7**	*N*-chloromethyl-*N*,*N*-dimethyltryptamine	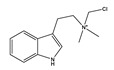	**C21**	desmodol	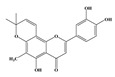
**C8**	*N*,*N*-dimethyltryptamine *N*^12^-oxide	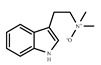	**C22**	caudatan C	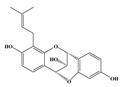
**C9**	*N*,*N*-dimethyltryptamine	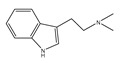	**C23**	citrusinol	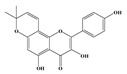
**C10**	nicotinic acid	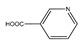	**C24**	yukovanol	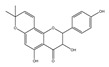
**C11**	ammothamnine	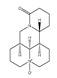	**C25**	caudatan A	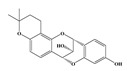
**C12**	loliolide	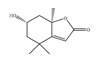	**C26**	3β-12-ene-3, 23, 28-triol	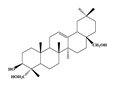
**C13**	salicylic acid	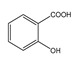	**C27**	soyasapogenel B	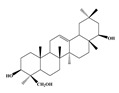
**C14**	ferulic acid	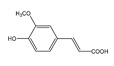	**C28**	(+)-5′-methoxyisolariciresinol-9-*O*-β-d-glucopyranoside	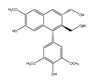

**Table 4:** Results of molecular docking studies of compounds **1**–**28** in the active sites of proteins (PDB ID 5F19, 2HZP, 3UQU, 3O0G, and 3UON) performed using Molegro Virtual Docker (MVD).

Compound	PTGS2	KYNU	BACE1	CDK5	CHRM2
Reference	−279.275	−71.135	−269.837	−157.913	−130.289
**1**	−109.826	−82.362	−131.327	−100.114	−124.923
**2**	−109.851	−81.0566	−125.875	−103.123	−113.16c
**3**	−101.917	−90.059	−97.622	−81.515	−102.699
**4**	−116.921	−112.357	−147.607	−125.264	−120.443
**5**	−66.7496	−59.008	−61.787	−83.45	−64.733
**6**	−87.8179	−85.553	−89.709	−71.066	−87.349
**7**	−97.7527	−80.036	−104.692	−87.507	−113.467
**8**	−96.806	−90.1924	−101.014	−82.776	−103.683
**9**	−95.682	−79.2415	−94.954	−83.71	−98.256
**10**	−87.768	−47.2166	−96.669	−73.057	−93.652
**11**	−83.11	−30.309	−78.291	−58.436	−77.146
**12**	−100.12	−61.922	−87.5	−77.206	−87.393
**13**	−74.49	−66.975	−67.683	−53.451	−66.144
**14**	−101.205	−89.905	−103.201	−84.782	−96.424
**15**	−91.99	−65.687	−101.422	−80.586	−103.666
**16**	−97.489	−64.878	−107.982	−84.402	−107.419
**17**	−113.147	−79.252	−131.154	−103.973	−126.566
**18**	−105.505	−82.359	−122.934	−97.775	−109.951
**19**	−121.982	−94.5721	−138.589	−113.973	−109.814
**20**	−118.492	−95.216	−128.598	−104.169	−124.998
**21**	−105.56	−80.545	−127.578	−102.149	−113.791
**22**	−101.112	−63.776	−126.693	−83.45	−117.44
**23**	−100.213	−64.122	−122.083	−96.788	−111.528
**24**	−99.225	−63.626	−119.269	−91.528	−110.426
**25**	−88.806	−55.989	−69.709	−73.446	−116.637
**26**	−89.21	−74.515	−95.219	−98.8	−67.588
**27**	−90.942	−52.395	−83.509	−101.319	−68.174
**28**	−118.408	−72.669	−136.226	−107.887	−133.31

## Supplementary Materials

The supplementary materials are available online.

## Targets Abbreviations

**Number**	**PDB ID**	**Targets**	**Abbreviations**
1	5F19	Prostaglandin G/H synthase 2	PTGS2
2	2HZP	Kynureninase	KYNU
3	3UQU	Beta-secretase 1	BACE1
4	3UON	Muscarinic acetylcholine receptor M2	CHRM2
5	3O0G	Cyclin-dependent kinase 5	CDK5
6	1aqw	Glutathione S-transferase	GSTP1
7	3DZU	Peroxisome proliferator activated receptor gamma	PPARG
8	5X68	Kynurenine 3-monooxygenase	KMO
9	1H8F	Glycogen synthase kinase-3 beta	GSK3*β*
10	4a79	Monoamine oxidase B	MAOB
11	5CXV	Muscarinic acetylcholine receptor M1	CHRM1
12	1SAC	Serum amyloid P-component	APCS
13	2YMD	5-hydroxytryptamine 4 receptor	HTR4
14	2FSO	Mitogen-activated protein kinase 14	MAPK14
15	1vzj	Acetylcholinesterase	ACHE
16	3IVH	β-Amyloid precursor protein	APP
