# Supplementary material for: Systems Pharmacological Approach to Investigate the Mechanism of Ohwia caudata for Application to Alzheimer’s Disease
Source: Molecules. 2019 Apr 17;24(8):1499. doi: 10.3390/molecules24081499 (PMC6515364; doi:10.3390/molecules24081499)
Supplement: Supplementary file 1 [file molecules-24-01499-s001.pdf]

Supporting information

Systems Pharmacological Approach to Investigate the Mechanism of *Ohwia caudata* for application to Alzheimer's disease

Yi-wei Sun[1,2], Yue Wang [1,2] and Da-li Meng[1,2,*]

[1] Key Laboratory of Structure-Based Drug Design and Discovery, Ministry of Education, Shenyang Pharmaceutical University, Shenyang 110016, PR China

[2] School of Traditional Chinese Materia Medica, Shenyang Pharmaceutical University, Shenyang 110016, PR China

Corresponding Authors:

*E-mail: mengdl@163.com

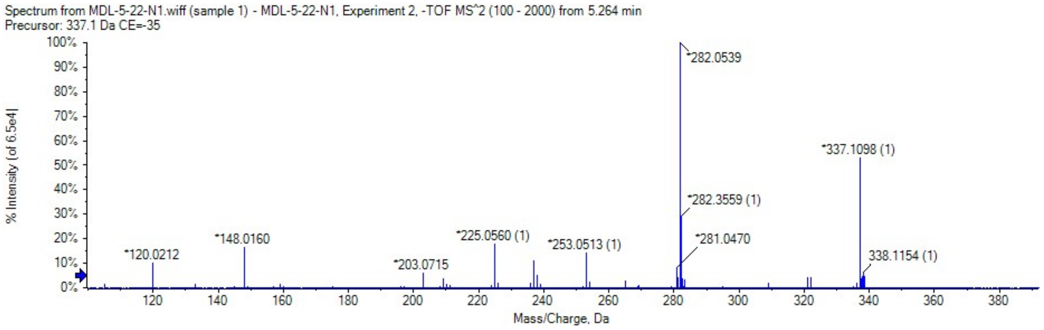

Figure 1. The HRMS Data of compound **1**

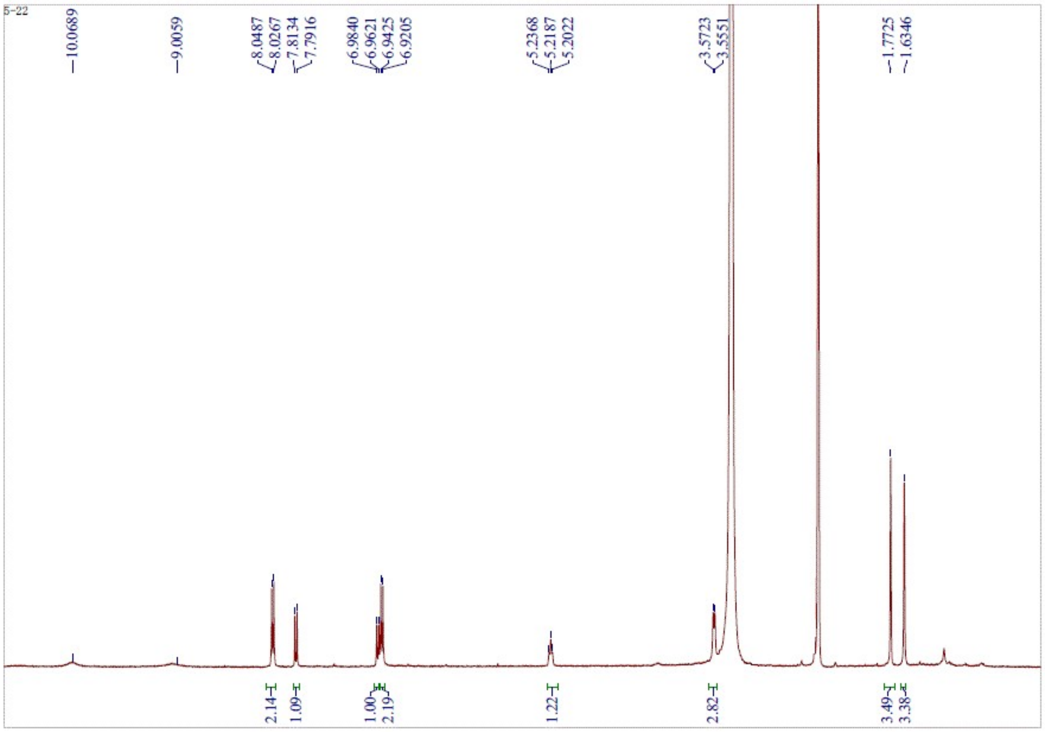

Figure 2. The ^{1}H NMR Spectrum of Compound **1** in DMSO-d_6 (600 Hz)

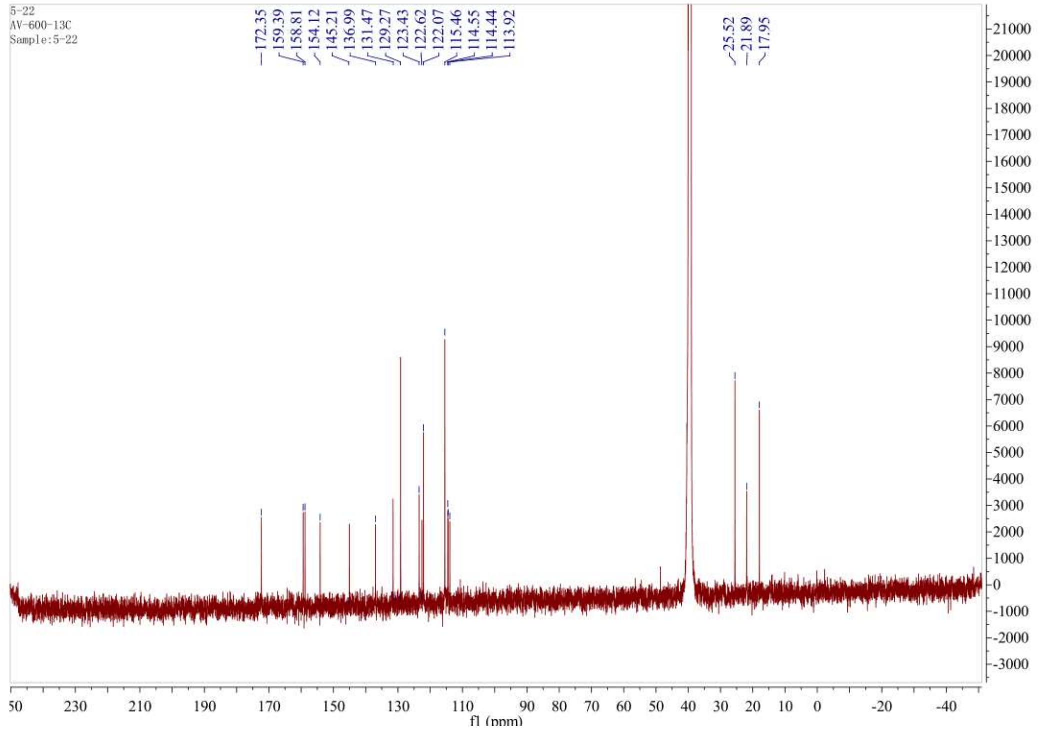

Figure 3. The ^{13}C NMR Spectrum of Compound **1** in DMSO-d_6 (150 Hz)

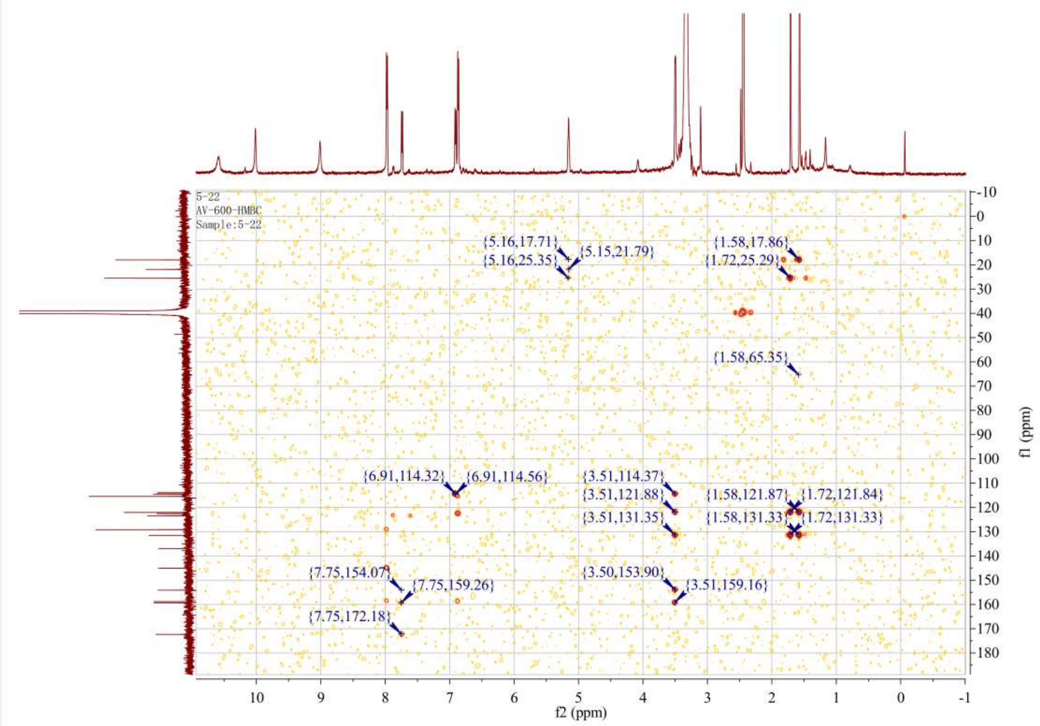

Figure 4. The HMBC Spectrum of Compound **1** in DMSO-d_6 (600 Hz)

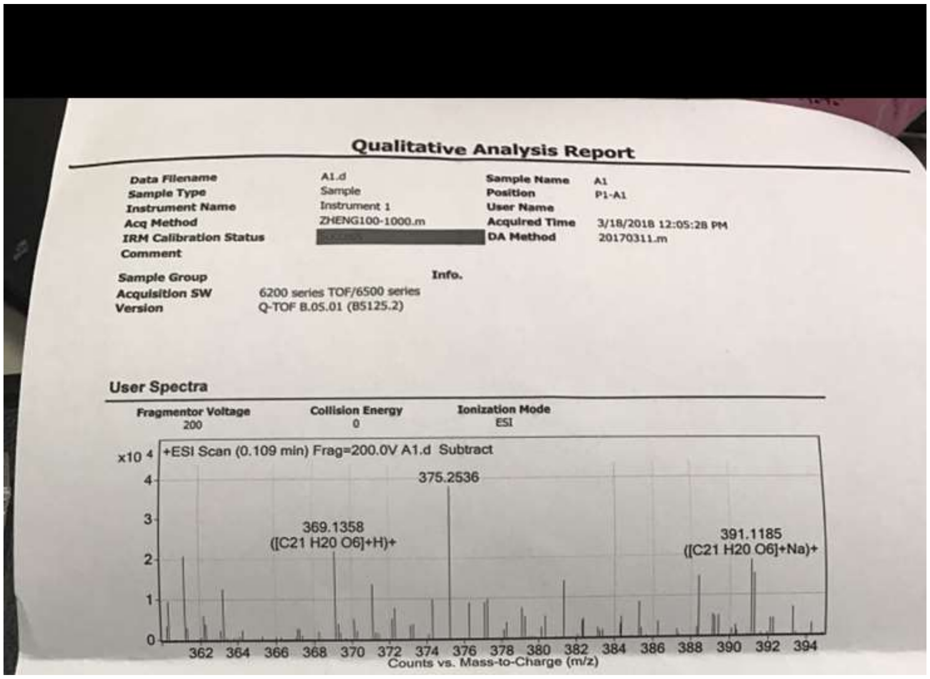

Figure 5. The HRMS Data of compound **2**

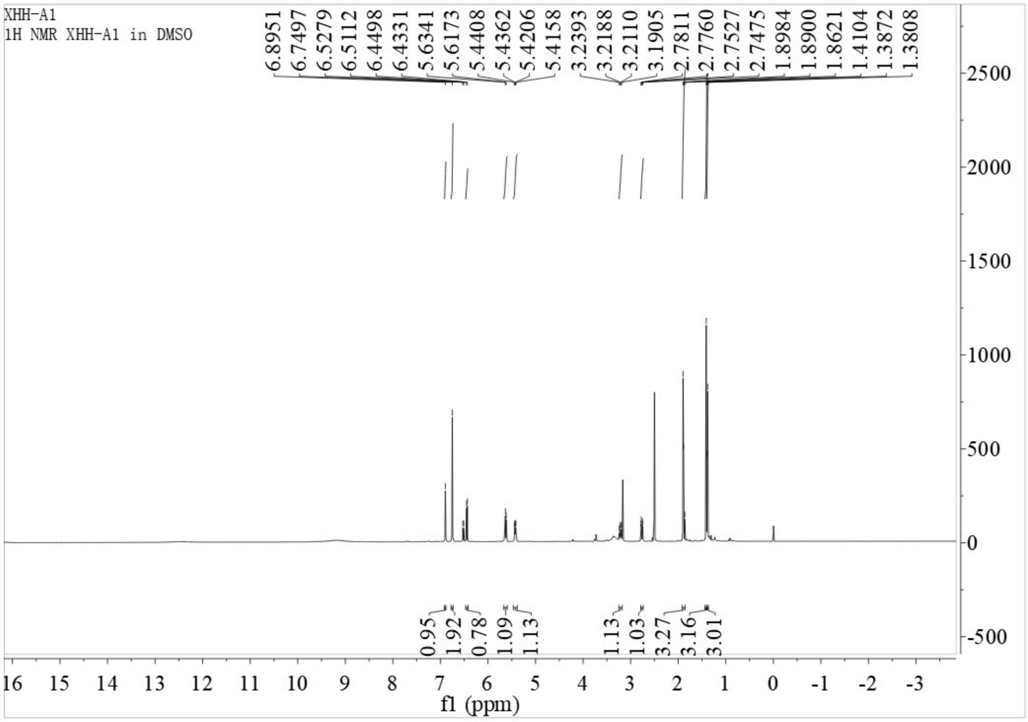

Figure 6. The ^{1}H NMR Spectrum of Compound **2** in DMSO-d_6 (600 Hz)

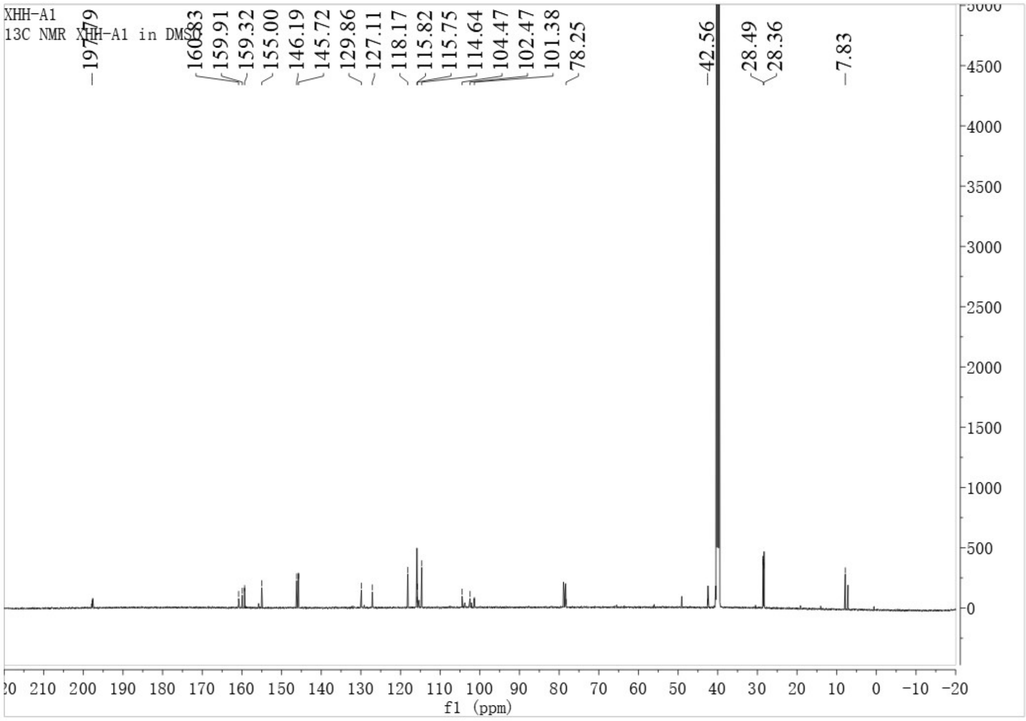

Figure 7. The ^{13}C NMR Spectrum of Compound **2** in DMSO-d_6 (150 Hz)

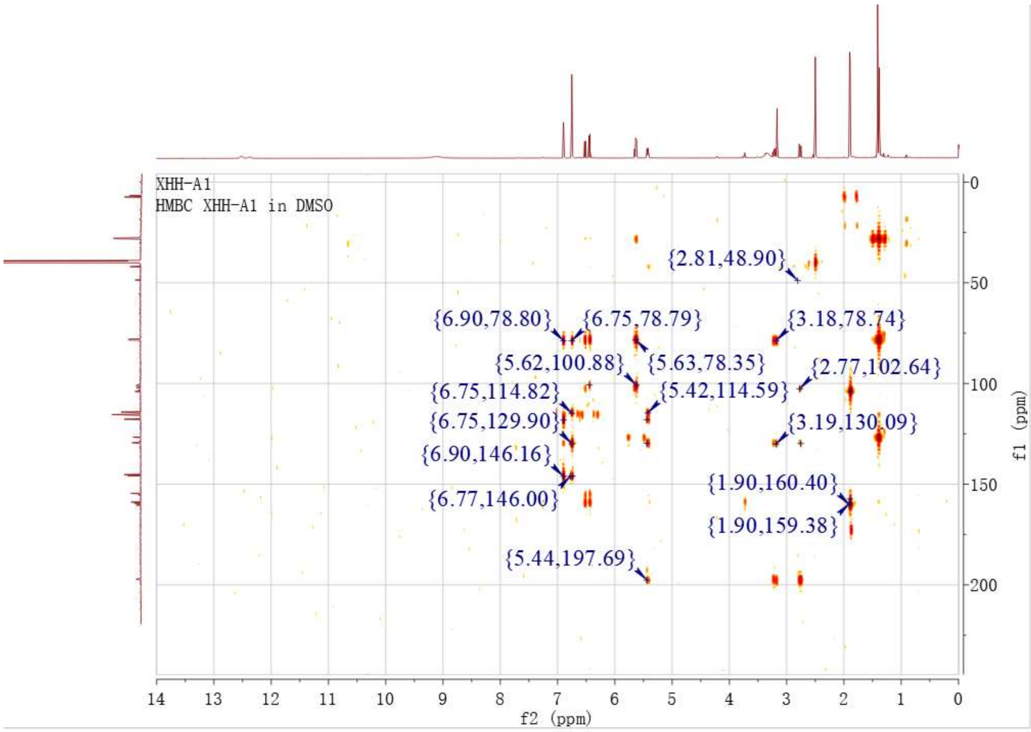

Figure 8. The HMBC Spectrum of Compound **2** in DMSO-d_6 (600 Hz)
